# The role of attachment in recovery after a school-shooting trauma

**DOI:** 10.3402/ejpt.v5.22728

**Published:** 2014-07-02

**Authors:** Tuija Turunen, Henna Haravuori, Raija-Leena Punamäki, Laura Suomalainen, Mauri Marttunen

**Affiliations:** 1 Hospital District of South Ostrobothnia, Seinäjoki, Finland; 2 Department of Psychology, School of Social Sciences and Humanities, University of Tampere, Tampere, Finland; 3 Department of Mental Health and Substance Abuse Services, National Institute for Health and Welfare, Helsinki, Finland; 4 Department of Psychiatry, Helsinki University Central Hospital, Peijas, Finland; 5 Department of Adolescent Psychiatry, Helsinki University Central Hospital, Espoo, Finland; 6 Department of Adolescent Psychiatry, Helsinki University Central Hospital, Helsinki, Finland

**Keywords:** Attachment style, school violence, dissociation, posttraumatic stress symptoms, posttraumatic growth

## Abstract

**Background:**

Survivors of life-endangering trauma use varying resources that help them to recover. Attachment system activates in the times of distress, and is expected to associate with stress responses, arousal regulation, and mental health.

**Objective:**

We examined the associations of attachment style with posttraumatic stress disorders (PTSD) symptoms and dissociative symptoms, and posttraumatic growth (PTG) among students exposed to a school shooting in Finland in a three-wave follow-up setting.

**Method:**

Participants were students (*M*_age_=24.9 years; 95% female) who were followed 4 (T1, *N*=236), 16 (T2, *N*=180), and 28 months (T3, *N*=137) after the shooting. The assessments included the Attachment Style Questionnaire, the Impact of Event Scale, part of the Adolescent Dissociative Experiences Scale and the Posttraumatic Growth Inventory.

**Results:**

Securely attached survivors had lower levels of posttraumatic stress and dissociative symptoms than preoccupied at T1 and T2 as hypothesized. At T3 survivors with avoidant attachment style had higher levels of intrusive and hyperarousal symptoms than those with secure style. Concerning PTG, survivors with avoidant attachment style scored lower in PTG at T3 than survivors with both secure and preoccupied style.

**Conclusion:**

Secure attachment style was beneficial in trauma recovery. A challenge to the health care systems is to acknowledge that survivors with preoccupied and avoidant attachment styles react uniquely to trauma, and thus need help in different doses, modalities, and timings.

School violence in the form of mass shootings causes feelings of horror, fear, and disbelief among students who suddenly lose their previously safe environment. Subsequently, survivors can suffer from various trauma-related symptoms such as acute stress disorder (ASD), posttraumatic stress disorders (PTSD) symptoms, depression, and anxiety. The severity of life danger and loss of close peers increases the risk for mental health problems (Hughes et al., 2011; Littleton, Grills-Taquechel, & Axsom, 2009; Suomalainen, Haravuori, Berg, Kiviruusu, & Marttunen, 2010). Not all survivors are similarly affected by traumatic events as each can have unique resources that contribute to recovery. These recourses are related, for example, to personality, social relations, and worldviews. The ways people cope with, make sense of, and regulate their emotions seem to play a crucial role in the success of recovery from trauma such as a school massacre (Boxer & Sloan-Power, 2013; Hughes et al., 2011). Attachment theory created by Bowlby (1969/1982) provides a good framework for understanding these individual differences in stress regulation and coping strategies when facing traumatic stress (Mikulincer & Shaver, 2010, p. 369–373).

According to attachment theory, the basis for the resources that promote or complicate the recovery after traumatic events is built in infancy when a child forms a unique communication pattern (attachment style) with his or her primary caregiver(s). This early relationship creates the conditions for a later sense of security or insecurity as infants learn how to regulate arousal and emotional reactions when distressed, and how to receive attention and support when threatened. These skills are internalized as working models and are generalized to other relationships later in life (Ainsworth, Blehar, Waters, & Wall, 1978; Bowlby, 1980).

Securely attached persons are confident that they will receive comfort, protection, and relief when facing trauma. They also have learned a variety of self-soothing and problem-solving skills that provide effective emotion regulating tools in distressing situations (Crittenden, 2000; Mickulincer & Shaver, 2010, p. 190). Avoidantly attached persons perceive help and support seeking as risky and uncomfortable, even if in a life-threating situation (Mickulincer & Shaver, 2010, p. 192–193). Those with preoccupied (ambivalent in childhood) attachment style face difficulties in regulating overwhelming feelings of fear, which may cause an overflow of threat-related thoughts toward helpless feelings (Crittenden, 2000; Sroufe, Carlson, Levy, & Egeland, 1999).

The exposure to trauma and stress later in life activates internalized attachment patterns, which play a critical role in the occurrence of subsequent trauma-related mental health problems, as well as in the choice of coping strategies and emotion regulation. Research has confirmed that securely attached survivors show low and insecurely attached high levels of mental health problems such as PTSD. Thus, secure attachment style is considered to be protective toward stress, whereas insecure attachment (both avoidant and preoccupied) is viewed as a risk factor to psychopathology (Fraley, Fazzarri, Bonnano, & Dekel, 2006; Mikulincer, Florian, & Weller, 1993). Fraley et al. (2006) studied the relationship between adult attachment and psychological adaptation in the context of 9/11 terrorist attacks (*N*=45). The results showed that survivors with avoidant or preoccupied attachment styles had more PTSD and depression symptoms than those with secure attachment style over time. A study by O'Connor and Elklit (2008) among Danish adults (*N*=328; 15–61 years) showed that secure attachment style was associated with low and avoidant attachment with high levels of lifetime and current PTSD symptoms. Finally, there is evidence from war-zones, mainly from the Middle East, that secure attachment style can protect both civilians and soldiers from PTSD and other mental health problems (Kanninen, Qouta, & Punamäki, 2003a; Mikulincer, Shaver, & Hores, 2006). However, a study among Israeli prisoners of war revealed that severe PTSD predicted insecure attachment style rather than vice versa (Solomon, Dekel, & Mikulincer, 2008). A study among Palestinian political prisoners (*N*=153) revealed that survivors with secure attachment style typically processed their trauma-related emotions in balanced ways, whereas survivors with avoidant attachment style relied narrowly on cognitive, and those with preoccupied attachment style on behavioral emotion regulation. The nature of emotion regulation in turn mediated the trauma impacts on mental health (Kanninen, Qouta, & Punamäki, 2003b).

Severe trauma exposure can lead to dissociative symptoms, which may also vary according to attachment style. In extreme life-threatening situations, such as mass killings, some victims attempt to protect their psychic integrity through dissociation that shields against overwhelming fear, pain, and feelings of helplessness (Van Der Hart, Nijenhuis, & Steele, 2006). Although dissociation may help survivors to protect themselves from the overwhelming emotions, research suggests that it associates with later mental health problems (Lensvelt-Mulders et al., 2008). There is some evidence that adolescents with secure attachment style show less dissociative symptoms than insecurely attached ones (Calamari & Pini, 2003; Nilsson, Holmqvist, & Johnsson, 2011). In their study of 162 students (16–24 years), Calamari and Pini (2003) found that insecurely attached students, particularly those with preoccupied style, had more dissociative symptoms such as amnesia and depersonalization than those with secure attachment style. Nilsson et al. (2011) report that dissociation was more common among insecurely attached students than among those with secure attachment style (*N*=568, 15–20 years). There is no earlier research on attachment style and dissociation in the context of a school shooting.

Trauma survivors do not only suffer but may also feel stronger, wiser, and more self-confident despite the horrifying experience. There is evidence that they often feel grateful for surviving and appreciate life and affiliation to other people, conceptualized as posttraumatic growth (PTG) (Taku, Cann, Calhoun, & Tedeschi, 2008). We found one study that examined the role of adult attachment style accounting for trauma victims’ capacity for beneficial transformation. Findings of a study among Palestinian political prisoners (*N=*275) suggest that survivors with secure attachment reported more PTG (i.e., personal strength, positive affiliation to others, and spiritual change) than prisoners with preoccupied attachment style (Salo, Qouta, & Punamäki, 2005). Interestingly, among prisoners with secure attachment style, severe exposure to torture even increased PTG, whereas among those with avoidant style the exposure was associated with very low levels of PTG.

## Background

There have been two school shootings in Finland in recent years. The first occurred in 2007 and the second, that is studied here, 10 months later in Kauhajoki in September 2008. In that second incident, a student of an educational institution in Kauhajoki shot nine of his classmates and his teacher. He threatened several other people causing fear and terror and severely damaged the premises before killing himself. The educational institution is a combination of a Vocational Education Centre and the University of Applied Sciences situated in Kauhajoki, a rural town of 14,000 inhabitants.

## Research objectives

The aim of this study is to analyze the association of attachment style with mental health outcomes and PTG among students exposed to a school shooting in Finland, 4 (T1), 16 (T2) and 28 (T3) months after the incident. Our hypothesis was that survivors with secure attachment style report lower levels of PTSD and dissociative symptoms, and higher levels of PTG than survivors with insecure-avoidant and insecure-preoccupied attachment style.

## Method

### Participants and procedure

**Fig. 1:**
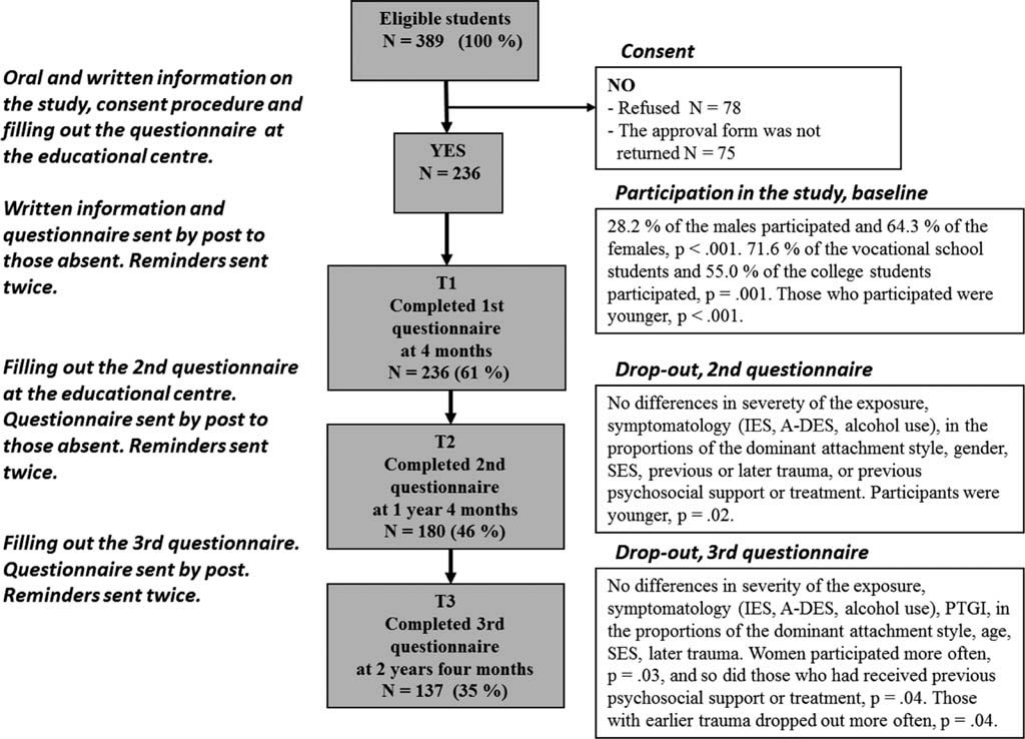
Participation of the Kauhajoki Educational Centre in the study after the school-shooting incident, flow chart.

The 2-year follow-up study was carried out at the National Institute for Health and Welfare in co-operation with the personnel of the educational institutions and the aftercare providers. The ethics committee of Hospital District of Southern Ostrobothnia, Finland, accepted study protocol. All students at the Kauhajoki Educational Centre who were present at the time of the incident were asked to participate in the study at three time points; 4 months (T1), 16 months (T2), and 28 months (T3) after the school shooting as described in Fig. 1. Of the 389 students, 60.7% (*N*=236) agreed to participate and completed the questionnaires at T1, 20.1% (*n*=78) declined, and 19.5% (*n*=76) could not be reached (Fig. 1). Mean age of the participants was 24.9 (SD=10.2), median age was 21, and some 20% of the participants were over 30 years of age (studied parallel to working, studies supported by employment services). Of those participating at T1, 180 (76.3%) participated also at T2 and 137 (58.1%) at T3. The severity of exposure to the school shooting and symptomatology was not associated with dropping out from the study (Fig. 1). Characteristics of the participating students are shown in Table 1.

**Table 1:** Sociodemographic and clinical characteristics of the students at the Kauhajoki Educational Centre participating in the study

Characteristic	*n*	%
Gender		
Male	11	4.7
Female	225	95.3
Age at the incident, M (SD)	24.9	(10.2)
SES		
Entrepreneur	20	9.4
Upper middle class	23	10.8
Lower middle class	51	23.9
Working class	92	43.2
Student	26	12.2
Other^a^	1	0.5
Living arrangements		
With both biological parents	60	25.4
With one biological parent	20	8.5
With spouse	105	44.5
Alone or other arrangements^b^	51	21.6
Received previous psychosocial support	33	14.0
Previous psychological treatment	26	11.3
Level of exposure		
Mild	43	18.2
Moderate	71	30.1
Significant	102	43.2
Severe	11	4.7
Extreme	9	3.8

*Note*: SES=socioeconomic status.

a Housewife or pensioner.

b With an adult other than a guardian, or with a child.

### Measures

The severity of trauma exposure was based on the level of threat to life and losses suffered (Suomalainen et al., 2010). Participants answered *yes* or *no* to 19 questions concerning their experiences during the shooting incident (e.g., “I saw the perpetrator,” “I saw someone get shot,” “I lost a good friend/friends”). Three questions also had space for additional comments. The answers were categorized into five classes indicating the severity: Mild, Moderate, Significant, Severe, and Extreme exposure. “Mild exposure” was rated when the student was not at the school building at the time of the shootings and “Moderate” exposure when the student was evacuated from the building, without being in direct danger of life and did not lose any acquaintances. “Significant exposure” was rated when the student faced danger of life and had to act to escape the shooter or had to hide, or the student saw dead or wounded bodies or lost acquaintances; “Severe exposure” was rated when the student was near mortal danger or saw somebody threatened with a gun or lost a friend(s) or some other significant person; and “Extreme exposure” was rated when the student was in mortal danger or saw somebody being shot and killed or she/he lost a family member.

Previous and later traumatization was assessed by a structured questionnaire. Participants answered *yes* or *no* accordingly to their previous experiences of traumatic incidents such as traffic accidents, natural disaster, witnessed or experienced violence, and provided the time of the incident. Answers were dichotomized: 0=no previous trauma, 1=one or more previous traumas.

Attachment Style was measured by items from the Attachment Style Questionnaire (ASQ), by Feeney, Hanharan, and Noller (1994). We used a short version that consists of 15 descriptions of how people typically feel in close relationships. Participants estimated items on a 1–6 Likert scale (1=strongly disagree, 6=strongly agree). The sum variables were formed following Feeney et al. (1994), and they depicted secure attachment style (five items, e.g., “I find it easy to trust others”; “I find it relatively easy to get close to other people”), avoidant attachment style (five items, e.g., “I worry about people getting too close”; “Achieving things is more important than building relationships,” and preoccupied attachment style (five items, e.g., “Other people often disappoint me”; “I worry that others won't care about me as much as I care about them”). Sum scores were calculated for the three attachment styles, showing sufficient internal consistency (Cronbach's α-values were 0.77 for secure, 0.70 for avoidant, and 0.70 for preoccupied attachment styles).

The three factor scores were also calculated with the 16-month follow-up material. Sum scores varied significantly between the predominant types of clusters in an almost similar manner to T1. Table 2 shows that the attachment style scores were similar at T1 and T2, indicating stability across time. Test–retest type of correlation analysis was performed to further test constancy of the attachment style. Interclass coefficient for the secure items was 0.76, *p*=0.21, for the avoidance items 0.71, *p*=0.08, and for the preoccupation items 0.71, *p*=0.16.

**Table 2:** Sum scores of the Attachment Style Questionnaire (ASQ) by predominant attachment style clusters

	Type of predominant attachment style			
		
ASQ sum scores T1, 4 months	Secure *n*=99	Avoidant *n*=80	Preoccupied *n*=50	*F*
Secure items^a^				
M (SD)	21.4 (3.4)	19.5 (2.9)	19.7 (3.2)	9.39*
Min–Max	11–29	10–28	14–29	
Median	22	20	19	
Avoidant items^b^				
M (SD)	11.5 (2.2)	16.4 (2.3)	13.8 (2.8)	132.19***
Min–Max	6–16	12–23	9–22	
Median	10	16	13.5	
Preoccupied items^c^				
M (SD)	10.6 (2.3)	13.4 (2.1)	17.5 (2.6)	148.58***
Min–Max	5–16	9–19	13–23	
Median	11	13	17	
T2, 16 months	Secure *n*=79	Avoidant *n*=62	Preoccupied *n*=37	

Secure items^d^				
M (SD)	21.9 (3.0)	20.3 (3.3)	19.4 (2.5)	10.50***
Avoidant items^e^				
M (SD)	12.1 (3.1)	15.5 (3.4)	13.9 (3.1)	18.64***
Preoccupied items^f^				
M (SD)	11.2 (2.8)	12.9 (2.9)	15.2 (3.6)	23.03***

a The Shceffé's post hoc analysis confirmed that the mean of the secure items was higher in the secure cluster than in the two insecure clusters.

b The mean of the avoidant items was highest in the avoidant cluster and lowest in the secure cluster.

c The mean of the preoccupied items was highest in the preoccupied cluster and lowest in the secure cluster.

d The mean of the secure items was higher in the secure cluster than in the two insecure clusters.

e The mean of the avoidant items was equally high in the avoidant cluster and preoccupied cluster and lower in the secure cluster.

f The mean of preoccupied items was highest in the preoccupied cluster and lowest in the secure cluster.

*** *p*<0.001.

Posttraumatic stress symptoms were measured by the Impact of Event Scale (IES) by Horowitz, Wilner, and Alvarez (1979) version IES-22 that consists of 22 questions on posttraumatic symptoms. Participants estimated items on scale 0=not at all, 1=rarely, 3=sometimes, and 4=often, based on their experiences during the previous week. The sum variables were formed depicting intrusive, avoidant, and hyperarousal symptoms. Sum scores for the total scale and the three subscales were calculated at T1, T2, and T3 and used as continuous variables. Good internal consistency among the total scale and the subscales was observed. Cronbach's α for the total PTSD symptoms was 0.94, for the IES-Intrusive 0.89, IES-Avoidance 0.85, and IES-Hyperarousal 0.87 at T1 (α-values were 0.95, 0.89, 0.90, and 0.85 at T2 and at 0.95, 0.89, 0.90, and 0.88 at T3, respectively).

Dissociative symptoms were assessed by The Adolescents Dissociative Experience Scale (A-DES) based on the Dissociative Experience Scale (DES) by Bernstein and Putnam (1986). The high correlation between these two versions has been reported by Armstrong, Putnam, Carlson, Libero, and Smith (1997). Tolmunen et al. (2007) have assessed dissociation in a sample of Finnish general population of adolescents aged 13–18 years (*N*=4,019) using A-DES. The mean A-DES score of 0.88 in the whole sample was lower than that in previous studies in other countries (Tolmunen et al., 2007). The A-DES originally has 30 questions. For practical reasons, we had to cut down items to nine, involving items on amnestic dissociation (2), depersonalization (3), derealization (1), hearing voices (1), and acting like someone else (2). The participants answered on a 0–10 Likert scale (0=never, 10=always) how frequent the symptom was. The mean sum score of the items was used for the analyses. Cronbach's α was 0.86 at T1, 0.80 at T2, and 0.88 at T3.

PTG was measured by the Posttraumatic Growth Inventory (PTGI; Tedeschi & Calhoun, 1996). The original PTGI has 21 items that involve dimensions of affiliation to others (seven items), new possibilities (five items), personal strength (four items), spiritual change (two items), and appreciation of life (three items) (Taku et al. 2008). Two items (one from affiliation to others and one from new possibilities) had to be omitted from the Finnish version due to very low loadings. The participants rated the questions on a 1–5 Likert scale (“I did not experience this change” to “I experienced this change to a very great degree”). The total sum score (Cronbach's α=0.93 at both T2 and T3) and five subscales of relating to others (α=0.84 at T2), new possibilities (α=0.79), personal strength (α=0.80), spiritual change (α=0.91), and appreciation of life (α=0.79) were applied in this study.

### Data analysis

The distributions of variables were presented as percentages for categorical variables and means (M) and standard deviations (SD) for continuous variables. To analyze how the attachment style associated with trauma-related symptoms and PTG, analysis of variance (ANOVA) with Scheffé's post hoc analysis was used. The three-class attachment cluster variable was the independent and PTSD, dissociation, and PTGI with subscales were the dependent variables. Factor analysis with the ASQ items was performed with principal component extraction method and rotated with Varimax method with Kaiser Normalization. In order to identify attachment clusters, hierarchical cluster analyses were performed with Ward's method to decide the appropriate number of the clusters to be formed. *K*-means cluster analysis was used to assign the studied individuals into the different cluster groups representing their dominant attachment style. The secure attachment style was compared separately with the two different types of insecure attachment styles (avoidant=1; secure=0; preoccupied=1), as a potential risk or protective factor on posttraumatic symptoms (IES) using multivariate linear regression analyses. Those background variables that associated with the symptoms were included as covariates (previous traumatization: no=0, yes=1; exposure: mild=1 to extreme=6; previous psychosocial support or psychological treatment: no=0, yes=1; and age). Socioeconomic status and living arrangements did not associate with the symptoms and were not included in the final model. Gender could not be analyzed due to low numbers of men in the sample.

In the analyses, two-tailed significance levels<0.05 were chosen. All analyses were performed using SPSS 20.0. Scales with less than 15% of missing items were accepted for the analyses while missing items were replaced by the series mean.

## Results

### Identifying attachment styles

The result of factor analyses of the 15 ASQ items resulted in expected secure, avoidant, and preoccupied attachment dimensions. One item, “I find that others are reluctant to get as close as I would like” loaded equally on preoccupation and avoidance factors and was omitted from further analyses. Cluster analysis resulted in three attachment clusters, and participants were assigned accordingly to “Secure,” “Avoidant,” and “Preoccupied” dominant attachment styles (Table 2). The distribution of attachment styles in this sample was: 43% (*n*=99) for secure, 35% (*n=*80) for avoidant, and 22% (*n=*50) for preoccupied.

### Attachment styles and PTSD and dissociative symptoms

The means and SD of PTSD symptoms according to the attachment style are shown in Table 3. As hypothesized, the securely attached survivors had significantly lower levels of total PTSD symptoms than those with preoccupied attachment style at 4 months after the trauma (T1) and in the 16 months follow-up (T2). However, contrary to our hypothesis, the survivors with avoidant attachment style also showed significantly lower levels of PTSD symptoms than those with preoccupied attachment style at T1, and did not differ statistically from the securely attached survivors at T1 and T2. The survivors with secure and insecure attachment styles did not differ significantly in the total level of PTSD symptoms at T3 (28 months). Similarly to the total PTSD symptoms, the securely attached survivors showed lower levels of avoiding and hyperarousal symptoms than those with preoccupied style at T1 and T2. Again, the survivors with avoidant attachment style did not differ from those with secure style. However, concerning PTSD symptoms at T3 the survivors with avoidant attachment style showed higher levels of intrusive and hyperarousal PTSD symptoms than the survivors with secure attachment style.

**Table 3:** Means and standard deviations (SD) of Impact of Event Scale (IES), Adolescent Dissociative Experiences Scale (A-DES), and Posttraumatic Growth Inventory (PTGI) in the different attachment style groups at 4 months (T1), 16 months (T2), and 28 months (T3) and ANOVA statistics for attachment effects with post hoc analyses to conclude which of the groups differ from each other

	Type of predominant attachment style				
			
Measures	Secure *n*=99, 79, 64 M (SD)	Avoidant *n*=80, 62, 42 M (SD)	Preoccupied *n*=50, 37, 28 M (SD)	*F*	Post hoc analyses (Sheffé)
T1 IES-22	22.4 (20.7)	27.6 (21.8)	39.0 (25.7)	9.24***	Sec<Pre, Avo<Pre
T2 IES-22	13.1 (16.2)	18.7 (20.5)	24.8 (23.4)	4.73*	Sec<Pre
T3 IES-22	8.2 (13.3)	16.2 (21.7)	16.5 (19.6)	3.62*	
T1 IES-Intrusive	7.7 (7.7)	9.7 (8.3)	12.7 (9.8)	5.76**	Sec<Pre
T2 IES-Intrusive	5.0 (6.2)	7.1 (8.1)	8.3 (8.3)	2.87	
T3 IES-Intrusive	3.3 (5.3)	6.7 (8.9)	5.7 (6.1)	3.47*	Sec<Avo
T1 IES-Avoidance	8.8 (8.3)	10.5 (8.3)	16.3 (9.8)	12.66***	Sec<Pre, Avo<Pre
T2 IES-Avoidance	5.1 (6.9)	7.2 (9.0)	10.1 (9.9)	4.43**	Sec<Pre
T3 IES-Avoidance	3.5 (5.7)	5.7 (8.7)	6.8 (9.1)	2.34	
T1 IES-Hyperarousal	5.9 (6.8)	7.4 (7.2)	10.1 (8.3)	5.48**	Sec<Pre
T2 IES-Hyperarousal	3.0 (4.6)	4.5 (5.7)	6.4 (6.9)	4.88**	Sec<Pre
T3 IES-Hyperarousal	1.4 (3.5)	3.7 (5.9)	4.0 (6.1)	4.04*	
T1 A-DES	2.3 (3.8)	4.7 (6.5)	6.3 (11.2)	6.18**	Sec<Pre
T2 A-DES	1.5 (4.0)	2.5 (3.5)	5.7 (9.0)	8.00***	Sec<Pre, Avo<Pre
T3 A-DES	1.6 (6.0)	1.8 (3.3)	3.6 (6.8)	1.35	
T2 PTGI	53.9 (14.2)	51.7 (14.3)	57.6 (15.1)	1.80	
T3 PTGI	55.6 (15.7)	51.2 (13.5)	58.0 (13.2)	1.77	
T2 relating to others	18.8 (5.0)	17.7 (5.0)	20.6 (4.9)	3.64*	Avo<Pre
T3 relating to others	19.3 (5.6)	16.9 (5.0)	20.6 (4.2)	4.24*	Avo<Sec, Avo<Pre
T2 new possibilities	10.0 (3.5)	9.9 (3.4)	11.4 (3.6)	2.49	
T3 new possibilities	10.4 (3.7)	9.5 (3.6)	11.3 (2.7)	2.02	
T2 personal strength	11.1 (3.6)	10.8 (3.9)	11.1 (3.8)	0.16	
T3 personal strength	11.6 (4.1)	11.3 (3.4)	11.7 (3.8)	0.13	
T2 spiritual change	3.2 (1.8)	3.3 (2.1)	3.7 (2.3)	0.63	
T3 spiritual change	3.4 (1.8)	3.4 (2.2)	3.5 (2.2)	0.02	
T2 appreciation of life	10.7 (2.9)	10.0 (2.7)	10.9 (3.1)	1.19	
T3 appreciation of life	10.8 (2.9)	10.2 (2.5)	11.0 (2.6)	0.88	

*Note*: T1=(first) questionnaire at 4 months, T2=(second) questionnaire at 16 months, T3=(third) questionnaire at 28 months. Sec=secure predominant attachment style, Avo=avoidant predominant attachment style, Pre=preoccupied predominant attachment style.

* *p*<0.05

** *p*<0.01

*** *p*<0.001.

Table 4 reports the results of multivariate linear regression analyses and confirms that the insecure-preoccupied attachment style was significantly associated with total PTSD symptoms (IES-22, *β*=0.20, *p*=0.009) and avoidance symptoms (IES-Avoidance, *β=*0.28, *p*<0.001) at 4 months, T1. The association was non-significant for IES-Intrusive (*p*=0.080) and IES-Hyperarousal (*p*=0.115) symptoms. Similarly to ANOVA results, the avoidant attachment style had no significant association with PTSD symptoms at T1 or T2, but was significantly associated with the total PTSD symptoms (*β=*0.21, *p*=0.034), IES-Intrusive (*β=*0.21, *p*=0.035), and IES-Hyperarousal (*β=*0.22, *p*=0.026) symptoms at 28 months, T3. The covariant of the severity of trauma exposure had a significant effect on IES symptoms at T1 and T2, but the effect of exposure attenuated by T3.

**Table 4:** Multivariate regression for the posttraumatic symptoms measured by the Impact of Event Scale (IES) studying the effects of avoidant and preoccupied attachment styles compared to secure attachment on recovering from a school-shooting trauma

	Avoidant vs. secure				Preoccupied vs. secure			
		
	*R* ^*2*^	B	SE B	β	*R* ^*2*^	B	SE B	β
IES-22								
T1	0.22	5.17	2.93	0.12	0.29	5.04	1.90	0.20**
T2	0.16	3.47	3.05	0.09	0.29	2.43	1.82	0.12
T3	0.11	7.59	3.53	0.21	0.17	1.96	1.81	0.11
IES-Intrusive								
T1	0.21	1.95	1.11	0.12	0.23	1.28	1.73	0.14
T2	0.14	1.0	1.20	0.08	0.25	0.51	0.68	0.07
T3	0.11	3.08	1.44	0.21	0.15	0.39	0.65	0.06
IES-Avoidance								
T1	0.15	1.72	1.20	0.10	0.25	2.78	0.78	0.28***
T2	0.08	1.44	1.38	0.09	0.24	1.21	0.80	0.14
T3	0.08	2.38	1.48	0.16	0.17	0.77	0.81	0.10
IES-Hyperarousal								
T1	0.21	1.50	0.97	0.11	0.27	0.97	0.62	0.12
T2	0.23	0.83	0.82	0.08	0.28	0.72	0.53	0.12
T3	0.12	2.13	0.93	0.22	0.15	0.81	0.53	0.16

*Note*: T1=(first) questionnaire at 4 months, T2=(second) questionnaire at 16 months, T3=(third) questionnaire at 28 months. Age, previous traumatization, previous psychosocial support or psychological treatment and level of exposure were controlled for. Gender and later traumatization could not be analyzed due to low numbers of males and new traumas in the sample.

** *p*<0.01

*** *p*<0.001.

Concerning the association between the attachment styles and dissociative symptoms, as hypothesized, the survivors with secure attachment style had lower levels of dissociative symptoms than those preoccupied at 4 months. Again, the securely attached survivors did not report less dissociative symptoms than those with avoidant attachment style, which defeated that part of the hypothesis. At T2, both secure and avoidant attachments styles differed from the preoccupied (Table 3).

### Attachment style and PTG

Table 3 further reports the results of Posttraumatic Growth Inventory (PTGI), revealing that attachment style was not associated with the total PTGI scores at T2 and T3. Against our hypothesis, the survivors with avoidant attachment style had a lower level of PTG relating to (affiliation with) others than those with preoccupied attachment style at T2 and lower levels than those with secure and preoccupied style at T3. The securely attached survivors did not differ from those with preoccupied style.

## Discussion

We analyzed the role of attachment style in associating and predicting posttraumatic stress and dissociative symptoms, and positive growth among students exposed to a school-shooting trauma in Finland. As expected, the secure attachment style was more protective than preoccupied style toward PTSD and dissociative symptoms. However, concerning PTG, survivors with secure attachment did not differ from insecure-preoccupied, although those with insecure-avoidant style showed a very low level of growth. The vulnerability of the two insecure attachment types, avoidant and preoccupied, differed according to the time that had elapsed since the school-shooting trauma. The survivors with preoccupied attachment style reported higher levels of PTSD and dissociative symptoms 4 and 16 months after the trauma, whereas those with avoidant style did not differ from the securely attached. In the long run, however, more than 2 years post-trauma, the survivors with avoidant style suffered most of the intrusive and hyperarousal PTSD symptoms. Our findings suggest that persons with different attachment styles show unique patterns of recovery, which is important to acknowledge in planning and tailoring psychosocial support and treatment.

The attachment theory highlights that facing threat and life danger such as in a school shooting, activates the attachment-specific ways of coping, regulating overwhelming emotions and relying on others’ support (Bowlby, 1969/1982). The first measurement in this study took place 4 months after the trauma, and apparently survivors’ minds were still fresh with fears and horrors. The students with insecure-preoccupied attachment style were most vulnerable to PTSD and dissociative symptoms, which may be due to their tendency to accelerate their negative feelings, thus facing difficulties to regulate excessive arousal. At the 16 months follow-up, the survivors with preoccupied attachment style still had a higher level of PTSD and dissociative symptoms than the securely attached students. The lower symptom levels of securely attached students may be explained by their wide variety of effective self-soothing and problem-solving skills, and their ability to trust in other people's availability for help and emotional consoling (Crittenden, 1997, 2000). Securely attached survivors apparently appraised their psychological state accurately and were able to seek help if needed.

The survivors with preoccupied style typically kept the terrifying scene of the school shooting vividly in their minds for a long time, as it is habitual to them to maximize their feelings of loss and fear. The trauma survivors with preoccupied attachment style have been found to show especially intrusive PTSD symptoms, which is due to their difficulty to frame, control, and deal with overwhelming emotions (Kanninen et al., 2003b; Mikulincer et al., 2006). This was also the case in our study at 4 and 16 months after the trauma. Our findings concur with the study on survivors of the 9/11 terrorist attack that also found that the survivors with preoccupied attachment style showed the most severe PTSD in the 18 months follow-up (Fraley et al., 2006). Similarly to ours, cross-sectional studies by Calmari and Pini (2003) and Nilsson et al. (2011) showed that students with secure attachment style had lower levels of dissociation than those with preoccupied attachment style.

When two years had elapsed from the school-shooting trauma, students with insecure-avoidant attachment style showed higher levels of intrusive and hyperarousal PTSD symptoms than the securely attached, which accords with the hypothesis. Typically, survivors with avoidant attachment style try to suppress their threat-related emotions, deny experienced threat of life, and numb threat-related emotions (Crittenden, 2000; Mikulincer & Shaver, 2010). They are used to trust themselves as a source of support, and they may feel that seeking help in a traumatic situation is a sign of weakness. Their basic assumption is not to trust others, which in the aftermath of the school shooting may have led to isolation, fear of sharing experiences, and failure to seek adequate help. This explains why they would suffer from intrusive symptoms such as nightmares and flashbacks when a long time has elapsed since the trauma. As survivors with avoidant attachment style have difficulties expressing their emotions and their need for support, other people may fail to recognize their despair. In their study of recovery after terrorist attacks, Fraley et al. (2006) found that survivors with avoidant attachment style had a relatively high level of PTSD, but the symptoms were unrecognized by their family members and friends. Moreover, friends and peers thought that these trauma survivors were doing fine. This miscommunication may leave the course of symptom development invisible among persons with avoidant attachment style. When tailoring interventions and help, it is essential to keep in mind that survivors with avoidant attachment style have difficulties relating to others and asking for help, even if they are in great need.

The results of attachment style and PTG do not support the hypothesis that survivors with secure attachment style are the most successful in experiencing the possible positive sides of the trauma. Instead, survivors with insecure-preoccupied attachment style scored similarly to the securely attached survivors and higher than those with insecure-avoidant attachment style. It is noteworthy, that survivors with avoidant style showed very low growth in the PTGI dimension of relating to others. To gain a positive and growth-inducing experience after a tragedy requires support and sharing, and therefore survivors with avoidant attachment style are vulnerable as sharing with others is not a natural way for them (Crittenden, 1997; Kanninen et al., 2003a).

We were able to reach about two thirds of the exposed students. Yet, this proportion can be considered satisfactory considering the circumstances. The dropout at follow-ups was not dependent on the severity of trauma exposure or posttraumatic or dissociation symptoms. The majority of the students in the Kauhajoki Educational Centre are women (90%), which explains the female predominance in the sample. As a result, we could not examine the differences between male and female students, which might have given additional information about the studied phenomena. To avoid the questionnaire being excessively long, we were only able to use a limited number of items in some of the questionnaires (e.g., dissociative symptoms). Self-administered questionnaires may include reporting biases and are thus not as reliable as information from structured interview methods. However, this methodology allowed us to collect a large sample providing information about various types of mental health outcome. We have performed multiple testing on, for example, PTSD symptoms with subscales and different time points. This may increase the risk of chance capitalization, and caution should be taken not to overvalue the results presented. As we carried out the study as partners to the aftercare provision, the results served as screening those in need of intervention.

## Conclusions

It is a great challenge for the health care professionals to plan and tailor effective interventions for survivors of traumatic, life-endangering experience, such as a school shooting. Psychosocial support and clinical interventions should be implemented in an attachment-specific way, keeping in mind that the survivors with different attachment styles have unique ways of coping, arousal regulation, the expression of emotions, as well as preparedness to seek help.

The conclusion of our study is that students with secure attachment style have the most beneficial means to recover after a school-shooting trauma. Both insecure-preoccupied and insecure-avoidant survivors are vulnerable, but may need help in different doses, modalities, and timing. We argue that those with preoccupied attachment style express their distress openly and their despair is easy to recognize and support offered. The insecure-preoccupied students are vulnerable especially at the wake of the trauma. Instead, it can be problematic to reach survivors with avoidant attachment style who may have persistent posttraumatic symptoms, but are not expressing their distress or seeking help. Further research is needed on the attachment-specific help-seeking behavior as well as on the different kinds of support that match the needs of survivors with secure and insecure attachment styles.
