# Corrigendum

**DOI:** 10.3402/ejpt.v5.26871

**Published:** 2014-12-17

Regarding the article ‘The role of attachment in recovery after a school-shooting trauma’ by *Tuija Turunen, Henna Haravuori, Raija-Leena Punamäki, Laura Suomalainen and Mauri Marttunen*

Published in *European Journal of Psychotraumatology* 2014. Citation: European Journal of Psychotraumatology 2014, **4**: 22728 - http://dx.doi.org/10.3402/ejpt.v5.22728

Two errors were noticed in this article:

1) The measure part concerning Impact of Event Scale is incorrect

2) In Table 4, the stars indicating the beta-values at time point T3 are missing from the Secure-avoidant regression analysis after beta values IES-total (value .21), IES-intrusive (.21) and IES-hypoarousal (value .22)

The correct paragraph and table are displayed below.

Posttraumatic stress symptoms were measured by the Impact of Event Scale (IES) by Horowitz, Wilner, and Alvarez (1979) version IES-22 that consists of 22 questions on posttraumatic symptoms. Participants estimated items on scale 0=not at all, 1=rarely, 3=sometimes, and 5=often, based on their experiences during the previous week. The sum variables were formed depicting intrusive, avoidant, and hyperarousal symptoms. Sum scores for the total scale and the three subscales were calculated at T1, T2, and T3 and used as continuous variables. Good internal consistency among the total scale and the subscales was observed. Cronbach's α for the total PTSD symptoms was 0.94, for the IES-Intrusive 0.89, IES-Avoidance 0.85, and IES-Hyperarousal 0.87 at T1 (α-values were 0.95, 0.89, 0.90, and 0.85 at T2 and at 0.95, 0.89, 0.90, and 0.88 at T3, respectively).

**Table 4:** Multivariate regression for the posttraumatic symptoms measured by the Impact of Event Scale (IES) studying the effects of avoidant and preoccupied attachment styles compared to secure attachment on recovering from a school-shooting trauma

	Avoidant vs. secure				Preoccupied vs. secure			
	
	*R* ^*2*^	B	SE B	β	*R* ^*2*^	B	SE B	β
IES-22								
T1	0.22	5.17	2.93	0.12	0.29	5.04	1.90	0.20**
T2	0.16	3.47	3.05	0.09	0.29	2.43	1.82	0.12
T3	0.11	7.59	3.53	0.21*	0.17	1.96	1.81	0.11
IES-Intrusive								
T1	0.21	1.95	1.11	0.12	0.23	1.28	1.73	0.14
T2	0.14	1.0	1.20	0.08	0.25	0.51	0.68	0.07
T3	0.11	3.08	1.44	0.21*	0.15	0.39	0.65	0.06
IES-Avoidance								
T1	0.15	1.72	1.20	0.10	0.25	2.78	0.78	0.28***
T2	0.08	1.44	1.38	0.09	0.24	1.21	0.80	0.14
T3	0.08	2.38	1.48	0.16	0.17	0.77	0.81	0.10
IES-Hyperarousal								
T1	0.21	1.50	0.97	0.11	0.27	0.97	0.62	0.12
T2	0.23	0.83	0.82	0.08	0.28	0.72	0.53	0.12
T3	0.12	2.13	0.93	0.22*	0.15	0.81	0.53	0.16

*Note*: T1=(first) questionnaire at 4 months, T2=(second) questionnaire at 16 months, T3=(third) questionnaire at 28 months. Age, previous traumatization, previous psychosocial support or psychological treatment and level of exposure were controlled for. Gender and later traumatization could not be analyzed due to low numbers of males and new traumas in the sample.

* *p*<0.05

** *p*<0.01

*** *p*<0.001.

